# The Characterisation of the Craniofacial Morphology of Infants Born With Zika Virus; Innovative Approach for Public Health Surveillance and Broad Clinical Applications

**DOI:** 10.3389/fmed.2021.612596

**Published:** 2021-06-24

**Authors:** Ashraf Ayoub, Leonardo de Freitas Silva, Peter Mossey, Dhelal Al-Rudainy, Adriana Marques de Mattos, Idelmo Rangel Garcia Júnior, Alan Quigley, Xiangyang Ju

**Affiliations:** ^1^Scottish Craniofacial Research Group, Dental School, College of MVLS, University of Glasgow, Glasgow, United Kingdom; ^2^Araçatuba Dental School, Univ Estadual Paulista, Araçatuba, Brazil; ^3^Scottish Craniofacial Research Group, School of Dentistry, University of Dundee, Dundee, United Kingdom; ^4^Dental School, College of MVLS, University of Glasgow, Glasgow, United Kingdom; ^5^Orthodontic Department, College of Dentistry, University of Baghdad, Baghdad, Iraq; ^6^Roberto Santos General Hospital, Salvador, Brazil; ^7^UNESP School of Dentistry at Araçatuba, São Paulo State University, Araçatuba, Brazil; ^8^Department of Paediatric Radiology, Royal Hospital for Sick Children, Edinburgh NHS Lothian, Edinburgh, United Kingdom; ^9^Scottish Craniofacial Research Group, Medical Devices Unit, NHS Greater Glasgow and Clyde Glasgow, Glasgow, United Kingdom

**Keywords:** craniofacial morphology, zika, surveillance, 3D imaging, principal compenent analysis, head measurements

## Abstract

**Background:** This study was carried out in response to the Zika virus epidemic, which constituted a public health emergency, and to the 2019 WHO calling for strengthened surveillance for the early detection of related microcephaly. The main aim of the study was to phenotype the craniofacial morphology of microcephaly using a novel approach and new measurements, and relate the characteristics to brain abnormalities in Zika-infected infants in Brazil to improve clinical surveillance.

**Methods:** 3D images of the face and the cranial vault of 44 Zika-infected infants and matched healthy controls were captured using a 3D stereophotogrammetry system. The CT scans of the brain of the infected infants were analysed. Principal component analysis (PCA) was applied to characterise the craniofacial morphology. In addition to the head circumference (HC), a new measurement, head height (HH), was introduced to measure the cranial vault. The level of brain abnormality present in the CT scans was assessed; the severity of parenchymal volume loss and ventriculomegaly was quantified. Student's *t*-test and Spearman's Rho statistical test have been applied.

**Findings:** The PCA identified a significant difference (*p* < 0.001) between the cranial vaults and the face of the Zika infants and that of the controls. Spearman's rank-order correlation coefficients show that the head height (HH) has a strong correlation (0.87 in Zika infants; 0.82 in controls) with the morphology of the cranial vaults, which are higher than the correlation with the routinely used head circumference (HC). Also, the head height (HH) has a moderate negative correlation (−0.48) with the brain abnormalities of parenchymal volume loss.

**Interpretation:** It is discovered that the head height (HH) is the most sensitive and discriminatory measure of the severity of cranial deformity, which should be used for clinical surveillance of the Zika syndrome, evaluation of other craniofacial syndromes and assessment of various treatment modalities.

## Introduction

The sudden outbreak of the Zika virus infection in 2016 raised grave global concern. The World Health Organisation (WHO) declared international public health emergency to raise the awareness of this epidemic.

It also called for support in scaling up and strengthening the surveillance systems in countries with reported cases of Zika virus–related microcephaly and other associated neurological conditions^1^.

As of July 2019, a total of 87 countries and territories have had evidence of autochthonous mosquito-borne transmission of Zika virus (ZIKV), distributed across four of the six WHO regions (African Region, Region of the Americas, Southeast Asia Region, and Western Pacific Region) (1). According to a recent report by the World Health Organisation, “It is also possible that some of these countries have or have had transmission that has not yet been detected or reported. All areas with prior reports of ZIKV transmission have the potential for re-emergence or re-introduction” (2). The Zika epidemic was particularly severe in the northeast corner of Brazil, where 97% of the country's Zika infections occurred (2). This area also accounts for 28% of all births in Brazil (3).

The Zika virus is transmitted primarily by Aedes mosquitoes; it has been detected in the fluid of pregnant mothers and in the brains of neonates, which causes microcephaly and other abnormalities (4, 5). As a result, the head circumference (HC) of infants is now routinely measured to identify microcephaly. The HC is one of the WHO's child growth standards (6). Microcephaly is defined as having an HC of < 2 standard deviations below the mean^1^. To rapidly identify Zika birth defects, it is recommended that a baby's HC should be measured before 24 h of age regardless of their laboratory test results. However, ~20% of infants with a normal HC have been reported to develop microcephaly at a later stage (7). Interestingly, the literature confirms that about 20% of these cases had normal HC values (8).

We believe that the current diagnostic criteria for microcephaly, based on skull HC measurement, are non-specific and cast too wide a net to capture real cases. Here, we explore if new morphometric methods can improve the sensitivity and specificity of Zika virus surveillance, to enable the 20% of infants currently identified as being normal to be correctly diagnosed and to improve the accuracy of microcephaly screening to support its early recognition and the timely evaluation and support of affected infants. To do so, we phenotyped the craniofacial morphology of infants born with microcephaly due to Zika virus infection and explored the most reliable method for screening by measuring the head shape and size. We also explored the relationship between the craniofacial phenotype and the degree of brain abnormality in these infants using CT scan data.

## Methods

### Ethical Approval

Approval for the study was obtained from the Brazil ethics committee (CAAE 60031216.4.0000.5420); mothers were consented for data capture and analysis.

### Patient Recruitment

The study was carried out at Robert Santos General Hospital, Salvador, Brazil on 44 infants infected with Zika virus (aged 10–21 months) and on 44 age- and sex-matched controls of the same ethnic background and geographic region. The craniofacial morphology of all the cases was recorded using 3D stereophotogrammetry. The postnatal head CT scans of the 44 Zika cases were captured by a 16-section scanner (Siemens Healthcare Limited, Surrey, United Kingdom), which were analysed by an independent consultant paediatric radiologist. The preliminary surveillance of Zika cases in this study entailed measuring their HC with a measuring tape.

### 3D Image Capture

**Figure 1:**
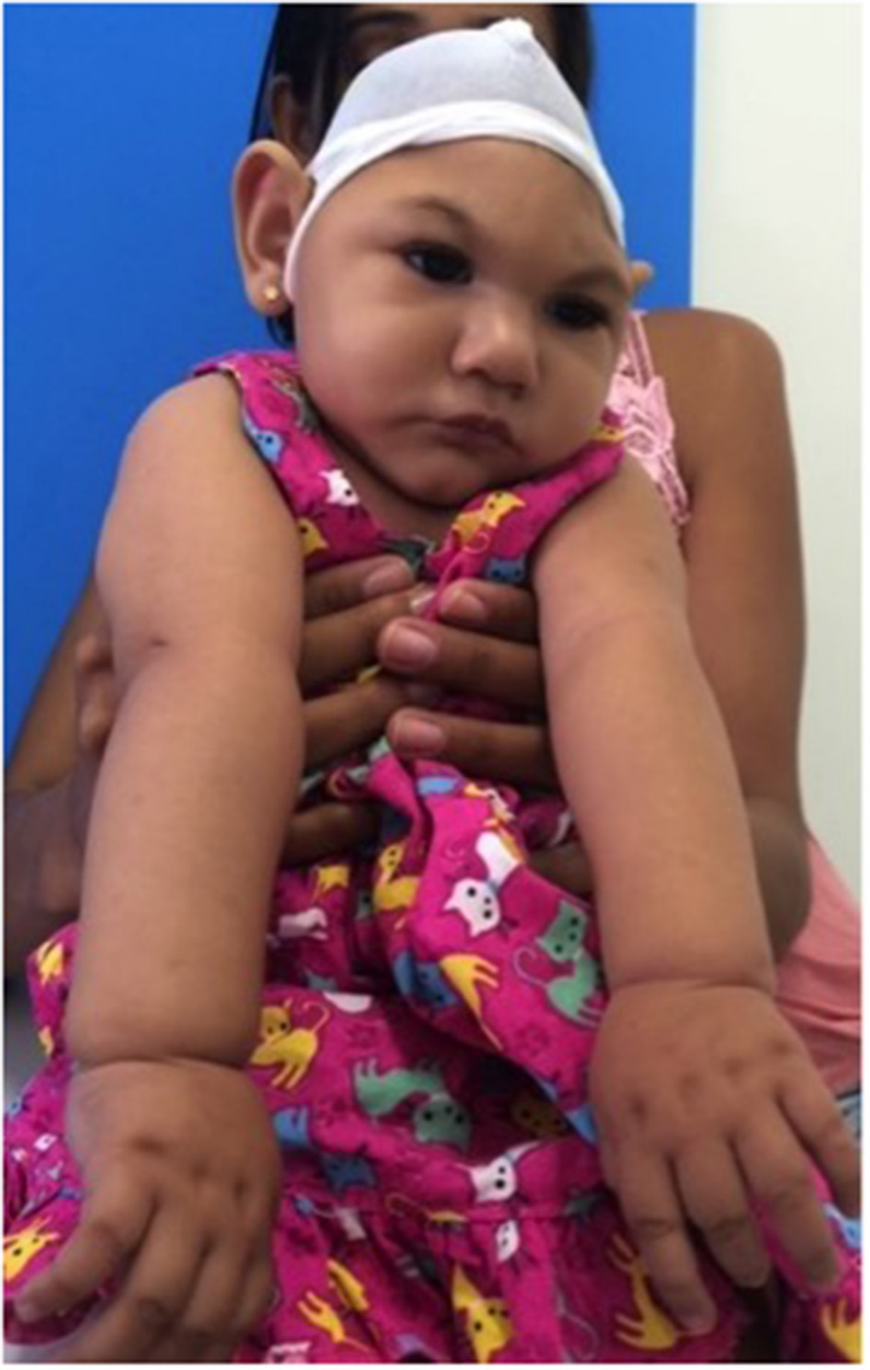
A mother holding her Zika infant in preparation for 3D facial imaging.

**Figure 2:**
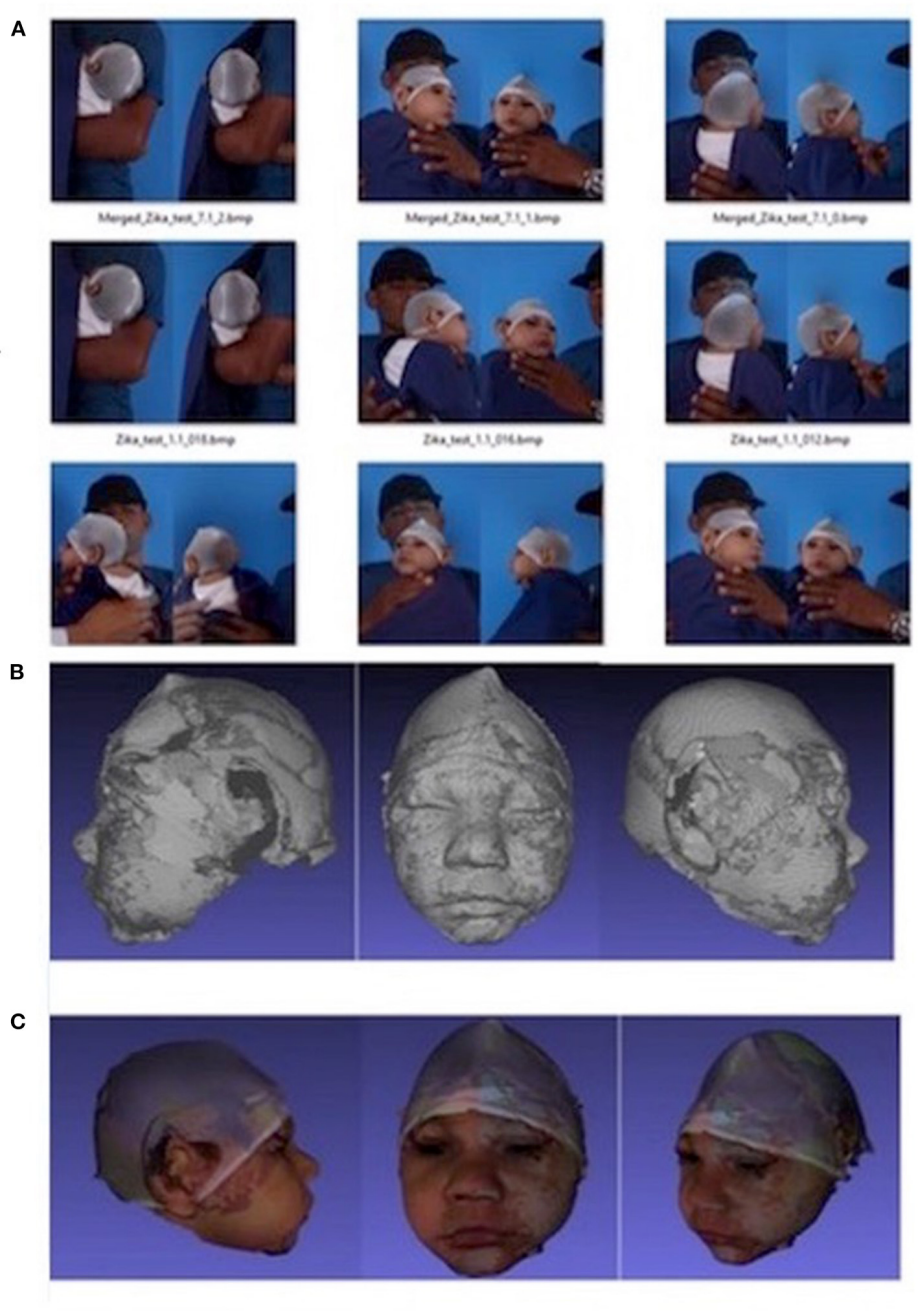
Capture of the head image using Di3D stereophotogrammetry imaging system. **(A)** 2D images captured, **(B)** 3D shapes reconstructed from **(A, C)** 3D models with shapes and textures **(A, B)**.

3D images were captured by a mobile 3D stereophotogrammetry imaging system (Di3D, Dimensional Image Ltd, Hillington, UK) (Figure 1). Due to the loss of muscle tone, the Zika infants were held by their mother (Figure 1) to allow the image capture, which took 1 ms. Multiple images of the face, including the right and left profiles, the vault of the skull and the back of the head, were captured (Figure 2). A custom-made disposable elastic head cap was used during image capture. 3D models of the craniofacial region were reconstructed using specially designed and developed Di3D software for this purpose. The multiple images captured of each case were assembled digitally to create a 360-degree model of the face and the cranium, which was saved in the Wavefront.obj file format (Figure 2).

### Morphometrics and Head Measurements

Generic facial meshes, which consist of mathematical masks of the face and the cranium made up of 7,461 indexed point “vertices” (7,145 vertices for the facial mask and 316 vertices for the cranial mask), were used for the craniofacial analysis. First, generic meshes were conformed to resemble the 3D characteristics of craniofacial morphology using a conformation software program (9). The accuracy of 3D image conformation has been previously evaluated and verified (10). This process was guided by a set of anatomical landmarks (33 landmarks for face conformation and 6 landmarks for vault conformation), followed by elastic deformation of the mesh. Centroid sizes were also calculated from the conformed 3D images of the Zika cases and controls.

Partial Procrustes analysis (PPA) is a mathematical method (11) that we applied to obtain an average mathematical face through the translation and the rotation without scaling for the accurate superimposition of the conformed meshes of the head vault and face of both Zika cases and controls. Generalised PA (GPA) was used to superimpose the conformed meshes of the cranial vault and face of both Zika and control cases with scaling (11, 12); this allowed variations in the craniofacial morphology of Zika cases relative to controls to be investigated regardless of the size differences involved.

Next, the GPA superimposed craniofacial meshes were analysed, and the principal components (PCs) were calculated using principal component analysis (PCA) to identify patterns of variation in the craniofacial morphology of Zika cases relative to controls.

**Figure 3:**
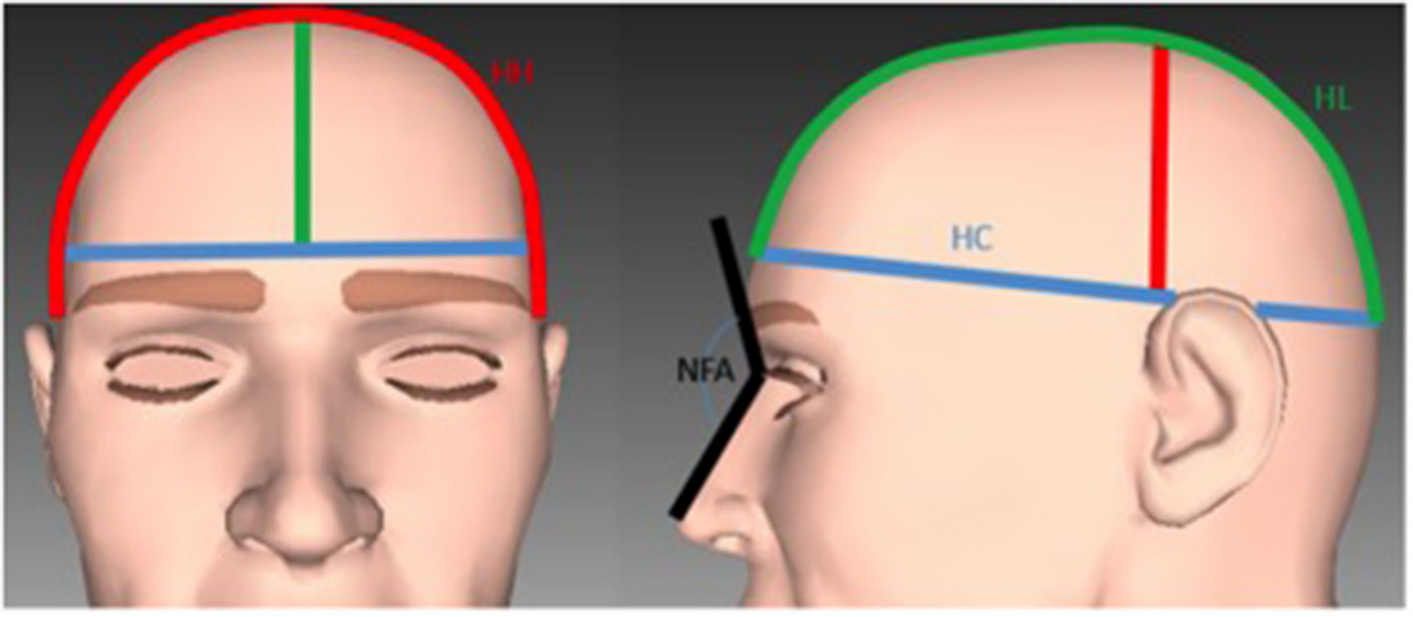
Illustration of the measurements used for the analysis of the cranial vault. Blue line: The head circumference. Red line: The head height. Green line: The head length. Black line: The naso-frontal angle.

The following additional measurements of the cranial vault were carried out (Figure 3):

a. The head circumference (HC), which is routinely measured from the most anterior point of the forehead to the most posterior point of the back of the head.
b. The head height (HH), the distance from ear to ear across the highest point of the vault of the skull.
c. The head length (HL), which measures the antero-posterior distance of the cranial vault, from the forehead, across the highest point of the cranial vault, to the most prominent point of the occipital bone.
d. The naso-frontal angle (NFA) between the three points: pronasal–nasion–glabella.

All measurements were repeated after 1 week by an experienced operator to assess the repeatability.

### Brain CT Scans

**Figure 4:**
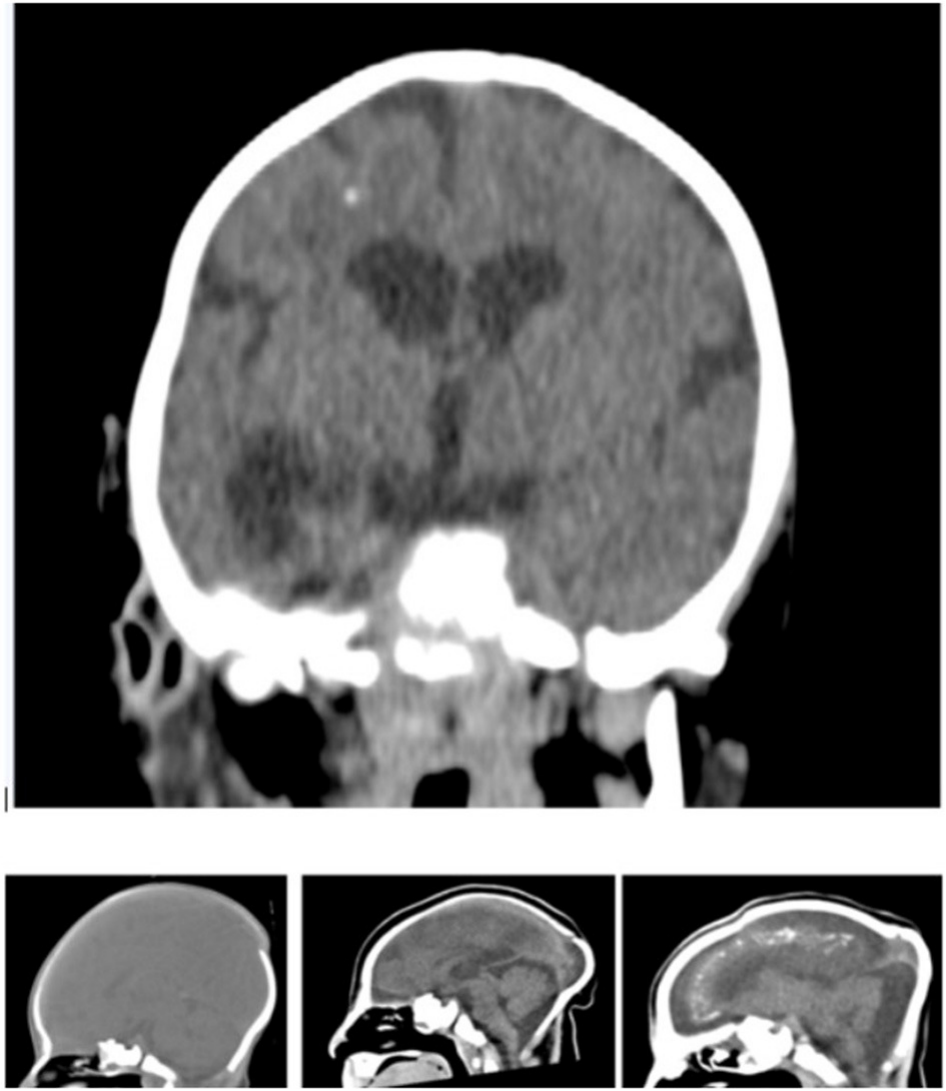
Coronal view of a non-contrast CT of an infant infected by Zika virus showing supratentorial volume loss and ventriculomegaly, pachygyria and foci of white matter calcification and sagittal images showing the normal and the moderate and severe parenchymal volume loss.

A consultant paediatric radiologist assessed the CT scans (Figure 4) using Horos^TM^ (https://horosproject.org/), a free open-source DICOM medical image viewer. The radiologist categorised the level of brain abnormality present in the CT scans, mainly the severity of parenchymal volume loss and ventriculomegaly, as being mild, moderate, or severe, according to Oliveira-Szejnfeld et al. (13).

### Statistical Analysis

Student's *t*-test (at the significance level of 0.05) was used to compare the scores of the PCs of the Zika cases with age-matched controls. The mean differences were assessed using Student's *t*-test, and the relationship between their craniofacial phenotype and the extent of brain abnormalities was assessed using Spearman's Rho statistical test.

## Results

All infants in the study received routine care, including neurologic examination and the recording of their weight and height. Abnormalities, including sleep problems, irritability, seizures, evidence of feeding or swallowing dysfunction, were identified in their clinical assessment. In the Zika cases of our cohort, epilepsy was present in 92.98% of the cases and refractory epilepsy in 29.09%. In addition, arthrogryposis was present in 12% of the Zika cases, as well as low vision (65.71%), dysphagia (70.00%) and sleep disturbance (60.87%). The main craniofacial characteristic was microcephaly with craniofacial disproportion, overlapping cranial sutures and redundant scalp skin. Neurologic abnormalities included spasticity, appendicular hypertonia, axial hypotonia, hyperreflexia and arthrogryposis.

Errors produced by the repeated measurement of the HC, HH (head height), and HL (head length) were <0.2 mm. The repeated landmarks' digitisation error was <0.5 mm.

**Figure 5:**
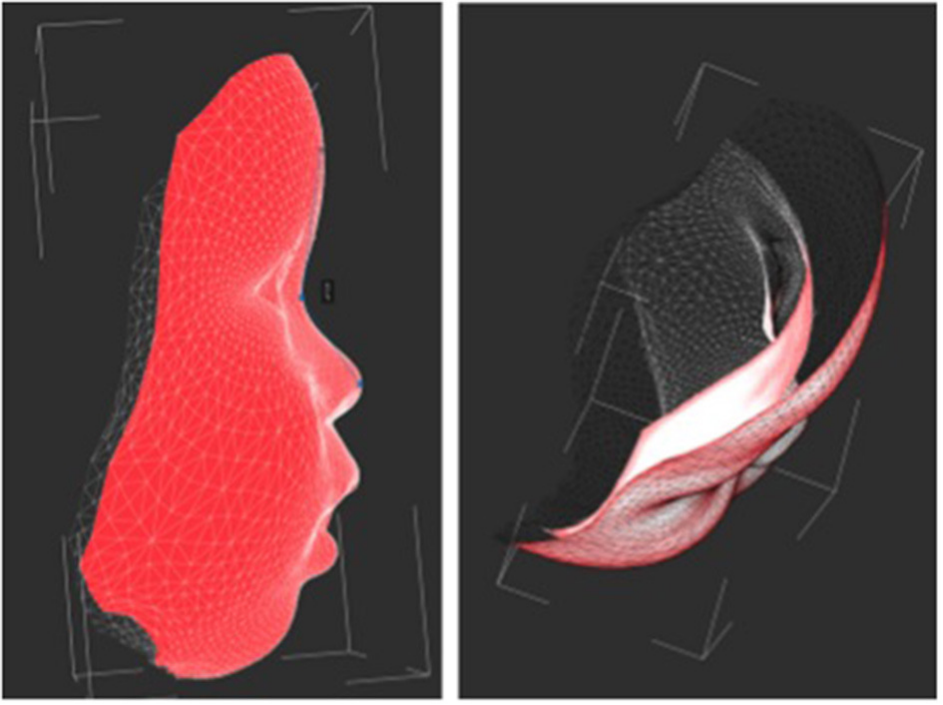
The average 3D facial shape of an age-matched healthy control (red mask) and with Zika (white). The images highlight the relatively prominent forehead seen in the control group.

Significant differences exist in the HC, HH, HL, and NFA (naso-frontal angle) of Zika cases relative to the age-matched controls (*p* < 0.001, < 0.05, Student's *t*-test). As calculated from the PPA superimposition analysis, the average shape of the head vault was significantly smaller in Zika cases relative to the controls (Figure 5).

The first 16 PCs explained 86% of the variation of facial shape, with individual contributions ranging from 24% (for PC1) to 1% (for PC16). The remaining PCs each contributed <1%. The two-sample *t*-test, followed by Bonferroni correction for multiple testing, revealed a high significant difference (*p* < 0.001) between the scores of the second PC in Zika cases relative to the controls; no other significant differences in the scores of the PCs were identified. The variations of −2α to 2α of the second PC of the cranial vault and the face proved that the head vault of the Zika group was under-developed (flat) compared to that of the controls, and the NFA of the Zika group was larger than that of the controls.

CT images were assessed by an independent consultant paediatric radiologist. The CT analysis revealed parenchymal volume loss in Zika patients that was mild in 25%, moderate in 22% and severe in 53% of the cases. It also revealed secondary ventriculomegaly (enlarged ventricles due to brain abnormalities in Zika patients), which was mild in 28%, moderate in 41% and severe in 28% of the cases. Brain ventricles were normal in 3% of the Zika cases. Abnormal cortical development was also evident in all Zika cases but with a wide range of variation. Calcification was predominantly present at the grey matter–white matter interface. Spearman's rank-order correlation coefficients showed a strong correlation (0.69) between parenchymal volume loss and ventriculomegaly, and significant correlations between parenchymal volume loss and HC (−0.53); parenchymal volume loss and HH (−0.48); and parenchymal volume loss and HL (−0.39). There was a significant correlation (−0.50, *p* = *0.0007*) between the severity of parenchymal volume loss and the centroid sizes of the cranial vault. This analysis suggests that the severity of parenchymal volume loss is negatively correlated to the size of the head vault of the Zika case. A significant correlation (−0.48, *p* = *0.0023*) was also detected between the severity of parenchymal volume loss and the scores of the second PC of the cranial vault.

Correlations were investigated between the shape and head measurements. In the Zika cases, a significant correlation (0.76) was detected between the score of the second PC of the cranial vault and the centroid size of the cranial vault. Significant correlations were also detected between the score of the second PC of the cranial vault and HC (0.66), HH (0.87), HL (0.80) and NFA (−0.48). In the control group, a significant correlation (0.58) was detected between the score of the second PC of the cranial vault and the centroid size of the cranial vault. Significant correlations were also detected between the score of the second principal component of the cranial vault and HC (0.33), HH (0.82), HL (0.62) and NFA (−0.42).

## Discussions

Zika virus infection is one of the most challenging global infectious epidemics in recent years (14). The WHO recommended and improved surveillance to better ascertain the incidence of ZIKV transmission (2). Therefore, we set out to explore a better head measurement for screening Zika microcephaly infants.

Our findings reveal statistically significant differences in the facial morphology and the cranial vault shape of the Zika cases relative to the healthy controls, as detected by the second PC of the PCA of the GPA superimposed shapes. A significant correlation between the head measurement and the cranial vault (microcephaly) was detected. Our results show that the most significant and sensitive measurement was the HH, which showed strong correlations (0.87 in Zika; 0.82 in control) with the scores of the second PC of the cranial vault, where the second PC represented the significant different shape characteristics of the cranial vaults in between the Zika cases and the control. In addition, a significant correlation between the magnitude of cranial deformity and the severity of parenchymal volume loss was detected. However, our analysis revealed that the HC and the second PC of the cranial vault were correlated at 0.66 in the Zika infants and weakly correlated at 0.33 in the control. The HC is a standard measurement that is routinely used to discriminate microcephaly from normal infants; however, our findings show that it is not the best measurement that correlated with distinctive cranial morphology.

It is current practice for paediatricians to measure the HC of infants and young children and to compare these measurements with the information provided by the National Centre for Health Case Statistics of the Centre for Disease Control and Prevention (15) and with WHO growth charts (6). However, it has been demonstrated that the HC measurement and the available norms lack sensitivity and specificity, and that significant variation in the HC exists among ethnic groups (2, 16). For example, Malay and Chinese infants have a significantly larger HC compared to Indian infants in the birth cohort. In addition, the existing method used to measure the HC (a measuring tape) fails to consider the shape of the cranial vault, and so it does not discriminate between microcephaly that is due to low birth weight and that due to craniofacial syndromes (17). The current method of HC measurement also does not describe the morphology of the vertex of the cranial vault nor does it discriminate between small size and a deformed skull.

A novel finding of this study is that a strong correlation exists between the distinctive cranial morphology of Zika infants and the head measurements of the HH and, to a lesser extent, the HL, which both provided sensitive measurements of the vertex of the cranial vault in Zika virus–infected infants. Strong correlations were detected between these two measurements (HH 0.87; HL 0.80) and the morphology of the cranial vault of the Zika infants. This is a logical finding; the new measurements encompass the shape of the cranial vault. The standard HC measure does not capture the morphological characteristics of the vertex of the vault of the skull, which is usually affected in a wide range of craniofacial syndromes (2). Franca et al. (3) have previously reported that 20% of Zika virus infections are associated with an HC that falls within the normal range, according to the international foetal and new-born WHO growth chart. It is important for paediatricians recognising the craniofacial phenotype of Zika cases to ensure their appropriate and timely evaluation and follow-up; our findings can facilitate achieving this important target (18). Based on the findings of this study, it is likely that a proportion of Zika-infected cases have not been detected early enough owing to the limitations of screening for microcephaly using the recommended WHO protocol of the HC alone. An interesting finding to emerge from this study is the increased naso-frontal angle (NFA) of Zika cases. Our results show that the NFA measure could discriminate the Zika virus cases from the control group. This has not been reported before and promises to be a robust and reliable measure to use when screening for microcephaly. Despite the fact that the new measurements improved the sensitivity of detecting Zika-related microcephaly, their specificity in differentiating various types of microcephaly has not been tested; this requires further investigation.

Computed tomography (CT) showed a wide range of abnormalities in Zika-infected infants, including subcortical calcifications, cortical thinning, abnormal gyral pattern, decreased myelination, absence of corpus callosum, cerebellar hypoplasia, closed anterior fontanel and cerebral atrophy (19, 20). Indeed, craniofacial dysmorphology reflected the severity of the associated parenchymal volume loss, which is a novel finding of this study. A previous attempt has been made to phenotype infants with Zika virus infection in three of the largest case series reporting 35, 48, and 104 infants with the congenital Zika virus syndrome, in which two-thirds of the infants had severe microcephaly (21, 22). In 75% of these cases, the occipital region was prominent. As such, the routine measurement of the HC from the frontal bone to occipital prominence, around the circumference of the skull above the ears, disregarding the vault of the skull, may not provide an accurate measurement of microcephaly because the prominence of the occipital bone would camouflage the underlying microcephaly. The new measurements presented in our study, including both the HH and the HL, would eliminate this potential source of measuring error when assessing the head size. The measurements provide a distinct improvement in the evaluation of the cranial vault assessment (23).

Our study demonstrates the significant association between the magnitude of parenchymal volume loss and the dysmorphology of the cranial vault. Since the severity of microcephaly is directly related to the underlying neurological damage, it could also be used as a prognostic indicator for the severity of the neurological deficit and the likely response to various modalities of treatment. The 3D image of the cranial vault would inform clinicians about the level of brain abnormalities, which would help to stratify the cases, according to the severity of the neural deficit, to a specialised management protocol.

We recognise that our study does have some limitations, which include the small sample size and the limited availability of CT scans to support the 3D imaging of craniofacial morphology. The study was limited to those diagnosed with Zika virus infection and microcephaly, and it remains uncertain if cases with Zika virus infection who did not have a traditional diagnosis of microcephaly were included in the cohort. The study was carried out at one centre where the outbreak of Zika infection was significant. Therefore, we have no information on the craniofacial characteristics of the Zika syndrome in other parts of Brazil.

The 3D stereophotogrammetry system has been applied on the capture and analysis of the craniofacial morphology (24–30); further mobile phone-based system (31) has been developed. In this paper, a 3D photogrammetry system has been applied on the analysis of the craniofacial morphology of infants born with Zika virus. The presented methods of 3D capture and analysis of the craniofacial morphology could be applied to a range of craniofacial anomalies; it facilitates monitoring of craniofacial development and the evaluation of the impact of various treatment approaches on cranial morphology. The 3D image system allowed the accurate, reliable and fast capture of facial morphology without exposing the infants to harmful radiation; it enabled detailed morphometric analysis of the Zika microcephaly, which separated the size from the morphology, which is crucial in this particular anomaly.

We hope that the findings of this study will aid the future screening and diagnosis of microcephaly across the globe where the detection of microcephaly is pertinent to the diagnosis, grading and management of other craniofacial syndromes. The developed algorithm lends itself to automated measurements, the development of robust diagnostic parameters and a wider application on other craniofacial anomalies. These findings cast doubt on whether the current WHO recommendation of measuring the HC is the best measure for the screening of microcephaly and other craniofacial anomalies. The routine measurements of the head size at the maternity units and labour wards should be re-evaluated, and the innovative measurements of our study should be considered. Further studies are required on new-borns before their application in neonatal and maternity wards.

## Conclusion

Significant correlations were detected between the craniofacial morphology and brain abnormalities. The HH is the most sensitive and discriminatory measure of the severity of the cranial deformity. The policy recommendation from this study is that the WHO should consider revising the guideline for the diagnosis of microcephaly from a circumferential to a transverse cranial measurement.

## Data Availability Statement

The raw data supporting the conclusions of this article will be made available by the authors, without undue reservation.

## Ethics Statement

The studies involving human participants were reviewed and approved by the Brazil ethics committee (CAAE 60031216.4.0000.5420). Written informed consent to participate in this study was provided by the participants' legal guardian/next of kin. The animal study was reviewed and approved by The Brazil ethics committee (CAAE 60031216.4.0000.5420). Written informed consent was obtained from the individuals, and minors' legal guardian/next of kin, for the publication of any potentially identifiable images or data included in this article.

## Author Contributions

AA: led the project, evaluation and interpretation of the findings, and drafting the manuscript. LF: 3D image capture and image integration, data processing, and analysis. PM: evaluation and interpretation of the findings and drafting the manuscript. DA-R: 3D image landmarking and conformation. AM: 3D image capture and clinical data analysis. IG: clinical data analysis. AQ: CD scan reading and clinical data analysis. XJ: 3D image morphometrical processing and analysis, evaluation and interpretation of the findings, and drafting the manuscript. All authors contributed to the article and approved the submitted version.

## Conflict of Interest

The authors declare that this study received a donated imaging system from Dimensional Imaging Limited. The donor was not involved in the study design, collection, analysis, interpretation of data, the writing of this article or the decision to submit it for publication.

## Footnotes

^1^https://www.cdc.gov/pregnancy/zika/testing-follow-up/zika-syndrome-birth-defects.html
