# Supplementary material for: The Characterisation of the Craniofacial Morphology of Infants Born With Zika Virus; Innovative Approach for Public Health Surveillance and Broad Clinical Applications
Source: Front Med (Lausanne). 2021 Jun 24;8:612596. doi: 10.3389/fmed.2021.612596 (PMC8264140; doi:10.3389/fmed.2021.612596)
Supplement: Supplementary file 1 [file Table_1.DOCX]

Varations of -2α (left) and 2α (right) of the second PC of the cranial vault added to the mean cranial valut (middle); and varations of -2α (left) to 2α (right) of the second PC of the face added to the mean face (middle)


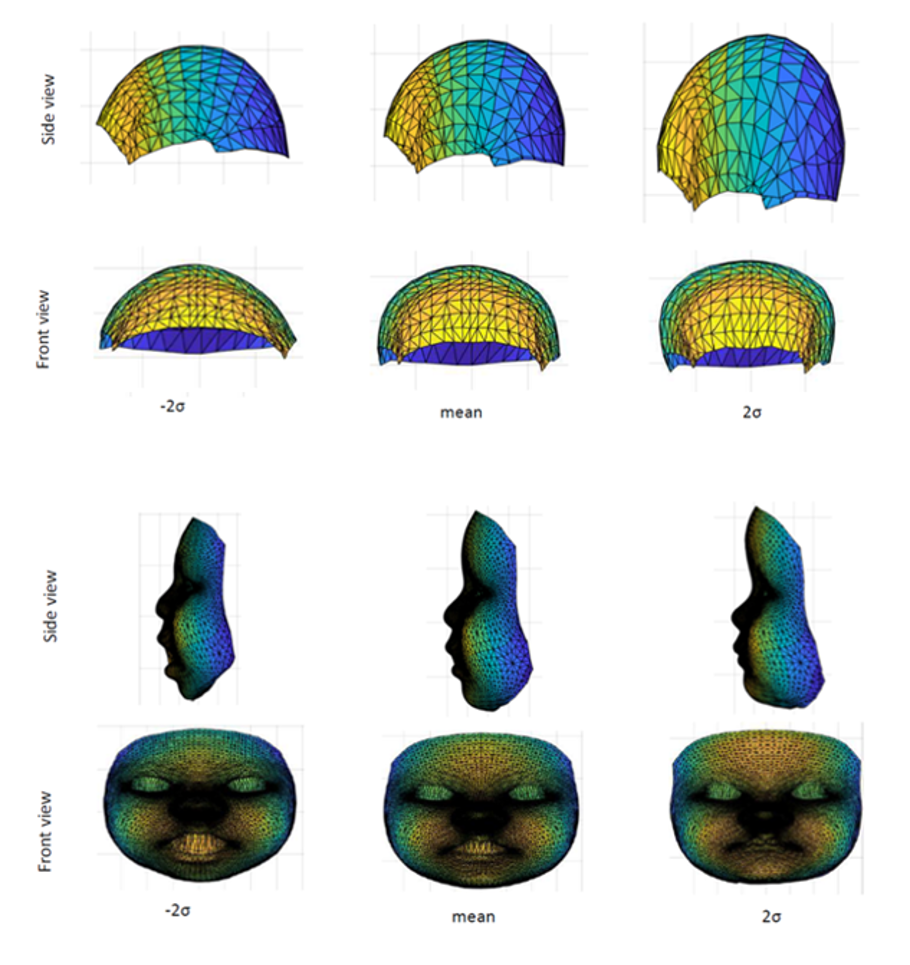


**Table.** The correlations between the principal components (PC) scores, head circumference (HC) and brain abnormalities in Zika cases and controls

| Significant correlations between PC scores, head measurements and brain abnormalities (Spearman’s Rho p<0.05, *p<0.01, **p<0.001) in Zika cases | | | | | | | |
| --- | --- | --- | --- | --- | --- | --- | --- |
|  | **Centroid V** | **HC** | **HH** | **HL** | **NFA** | **PVL** | **VM** |
| **PC2 Vault** | 0.76** | 0.66** | **0.87**** | 0.80** | -0.48* | -0.48* |  |
| **PC2 Face** | 0.34 |  |  |  |  |  |  |
| **Centroid Face** | 0.50* |  |  |  |  |  |  |
| **Centroid Vault** |  | 0.96** | 0.90** | 0.92** | -0.44 | -0.50* | -0.48* |
| **HC** |  |  | 0.87** | 0.86** | -0.35 | -0.53* |  |
| **HH** |  |  |  | 0.84** | -0.47* | -0.48* |  |
| **HL** |  |  |  |  | -0.44* | -0.39 |  |
| **PVL** |  |  |  |  |  |  | 0.69** |

| Significant correlations between PC scores, head measurements (Spearman’s Rho p<0.05, *p<0.01, **p<0.001) in controls | | | | | | |
| --- | --- | --- | --- | --- | --- | --- |
|  | **Centroid V** | **HC** | **HH** | **HL** | **NFA** |  |
| **PC2 Vault** | 0.58** | 0.33 | **0.82**** | 0.62** | -0.42 |  |
| **PC2 Face** |  |  |  |  |  |  |
| **Centroid Face** | 0.66* | 0.67** |  | 0.50 |  |  |
| **Centroid Vault** |  | 0.90** | 0.75** | 0.80** | -0.40 |  |
| **HC** |  |  | 0.54** | 0.69** |  |  |
| **HH** |  |  |  | 0.57** | -0.47* |  |
| **HL** |  |  |  |  |  |  |

**HH**: Head Height, **HC**: head circumference, **HL**: head length, **Centroid F**: centroid of the face, **Centroid V**: centroid of the vault of the skull, **PC2**: second principal component. **NFA**: nasofrontal angle. **PVL**: parenchymal volume loss. **VM:** ventriculomegaly.
